# The fungal volatile organic compound 6-pentyl-alpha-pyrone primes dwarf bean for enhanced drought tolerance

**DOI:** 10.3389/fpls.2026.1833542

**Published:** 2026-08-12

**Authors:** Jesús Francisco Echaide Aquino, John G. Hampton, Artemio Mendoza-Mendoza

**Affiliations:** 1 Department of Pest-Management and Conservation, Faculty of Agriculture and Life Sciences, Lincoln University, Lincoln, Canterbury, New Zealand; 2 Department of Agricultural Sciences, Faculty of Agriculture and Life Sciences, Lincoln University, Lincoln, Canterbury, New Zealand

**Keywords:** 6-pentyl-alpha-pyrone, drought tolerance, mitogen-activated protein kinase activation, fungal volatile organic compounds, priming, transcriptomics, trichoderma

## Abstract

Drought stress is a major constraint on crop production. While *Trichoderma* fungi are widely recognized for enhancing plant tolerance to abiotic stresses, the fungal molecules responsible for these protective effects remain poorly defined. In this study, we investigated the role of 6-pentyl-α-pyrone (6PP), a volatile compound produced by diverse *Trichoderma* spp., in modulating plant responses to water deficit. Dwarf bean (*Phaseolus vulgaris*) seedlings grown from seeds coated with a carrier polymer delivering 6PP were significantly more tolerant to drought stress than seedlings from seeds coated with the polymer alone (control), exhibiting increased leaf area, root length, chlorophyll content, and photosynthetic rate. To elucidate the mechanisms induced by this molecule, comparative transcriptome analyses were performed in the presence and absence of 6PP under both well-watered and drought conditions; only 49 DEGs were detected under well-watered conditions. In contrast, 476 expressed genes were identified in drought-stressed plants grown from 6PP-coated seeds, indicating substantial transcriptional reprogramming. 6PP treatment reprogrammed stress-responsive gene networks, including pathways associated with photosynthesis, carbohydrate metabolism, phosphorylation-mediated signaling, defense responses, and oxidative stress regulation. In addition, drought-stressed plants treated with 6PP exhibited enhanced MAPK phosphorylation, providing protein-level evidence for activation of phosphorylation-mediated signaling pathways associated with stress tolerance. Functional enrichment analyses further highlight secondary metabolite pathways, including those for phenylpropanoid and flavonoid biosynthesis, suggesting enhanced production of protective compounds and maintenance of photosynthetic activity under drought stress. Several stress-related genes were modulated prior to drought exposure, indicating a possible priming effect. Collectively, our findings identify 6PP as a potential signaling molecule contributing to enhanced drought resilience and provide new insights into the molecular basis of *Trichoderma*-mediated stress protection.

## 1 Introduction

Drought is a major constraint on agricultural productivity across all climate zones (Liu et al., 2025; Wang and Ren, 2025). Climate change has intensified both the frequency and intensity of drought events (Vicente-Serrano et al., 2022), particularly in major food-producing regions, resulting in substantial crop losses. For example, worldwide yield reductions of 25-40% for cereals, 20-85% for legumes, and 25-70% for root/tuber crops have been reported (Daryanto et al., 2017). Strategies to mitigate the impact of drought can be categorized as long-term (actions to reduce the vulnerability of water supply and increase the use of drought-resistant crops/cultivars) and short-term, such as improving irrigation efficiency and minimizing water loss (Wang and Ren, 2025). However, one approach often overlooked is plant priming, in which an early-stage stimulus enhances a plant’s tolerance to future stress events (Conrath et al., 2006; Martínez-Medina et al., 2017). Plant priming can be triggered by various stimuli, including microbial inoculants, phytohormones, and natural compounds (Conrath et al., 2006; Jatana et al., 2024; Kessler et al., 2006; Mauch-Mani et al., 2017). *Trichoderma* fungi are widely recognized for promoting plant growth and resilience, including enhancing tolerance to drought and other abiotic stresses (Bae et al., 2009; He et al., 2022; Jalali et al., 2017). Some of these protective effects have been attributed to the secretion of diverse bioactive metabolites by *Trichoderma* (Jalali et al., 2017). Among these, fungal volatile organic compounds (FVOCs) have emerged as promising priming agents, capable of modulating plant growth, development, and stress responses through mechanisms such as hormone modulation, transcriptional reprogramming, and systemic signaling (Hung et al., 2014). One of these FVOCs is 6-pentyl-α-pyrone (6PP), a lactone produced by some microorganisms and plants, including several *Trichoderma* species (Mendoza-Mendoza et al., 2024). Initially recognized for its antifungal properties, 6PP has also been shown to promote root elongation, modulate auxin signaling, and enhance plant growth under biotic stress (El-Hasan and Buchenauer, 2009; Garnica-Vergara et al., 2016; Kottb et al., 2015; Mendoza-Mendoza et al., 2024; van Zijll de Jong et al., 2023). However, whether 6PP alone can confer protection against abiotic stress, such as drought, remains largely unexplored.

Despite increasing evidence supporting the biological activity of 6PP, its use has as yet been limited to a small number of glasshouse and net-house experiments (Jiménez-Bremont et al., 2024; Mendoza-Mendoza et al., 2024) with application as a seed coating (Degani and Gordani, 2022), soil drench (El-Hasan and Buchenauer, 2009) or foliar spray (Pascale et al., 2017). Like other FVOCs, 6PP volatility presents challenges for stability and targeted delivery. To overcome this limitation, we incorporated 6PP into a polymer-based seed coating, which allows controlled release of the compound during germination and early seedling development (Pedrini et al., 2017). Using this approach, we investigated whether 6PP can prime dwarf beans to enhance drought tolerance and thereby induce abiotic stress protection. We hypothesized that 6PP-treated seeds would produce plants with improved physiological and molecular responses to drought. To test this, we assessed the protective effects of 6PP by assessing plant physiological and morphological traits under drought conditions and conducting transcriptomic analyses to uncover the molecular mechanisms underlying the priming response.

## 2 Material and methods

### 2.1 Plant material and seed sterilization

Fungicide-untreated dwarf bean seeds (*Phaseolus vulgaris* cv. Messi) obtained from Lefroy Valley Ltd, New Zealand were surface sterilized as follows: 100 g of seeds were placed in sterile distilled water for 2 minutes, then transferred to 70% ethanol for 2 minutes, then to 2% (V/V) sodium hypochlorite (NaOCl) for 2 minutes, followed by three rinses with sterile distilled water. The seeds were then individually placed on sterile filter paper, dried in a class II biosafety cabinet, and UV-treated before use.

### 2.2 Seed coating

The seed-coating polymer Seedworx ™ Bio Friendly 1 (Centor Oceania, Australia) was used as a carrier for applying the fungal volatile compound to the seeds. For every experiment, 1 milliliter of Bio Friendly 1 polymer (polymer) blend (polymer mixed with sterile water in a 1:1 ratio) was prepared according to the manufacturer’s instructions. When 6PP (Sigma-Aldrich, U.S.A.) was added to the blend, the amount of water was adjusted to maintain the 1:1 ratio between the polymer and the remaining components of the mixture. To homogenize the components the blend was transferred to 2 ml screw-top tubes and mixed using a FastPrep24 homogenizer (MP Biomedicals) at 5.5 m/s for 60 s. One milliliter of the polymer blend and 50 g of disinfected bean seeds were combined in a sterile 50 ml container and mixed manually for 5 minutes using circular and rotational motion to achieve a uniform coating. A preliminary dose-ranging assay was conducted to determine a biologically effective 6PP concentration under the experimental conditions used in this study. Based on the responses observed in this screening experiment, 6PP concentrations were increased from the initial micromolar range to the millimolar range for the main experiment. The selected concentrations were 75 mM, 114 mM, and 228 mM, which produced the clearest and most consistent physiological responses. The control treatment consisted of the polymer and water blend (1:1, v/v) without 6PP, while the 6PP treatments were the polymer plus water and the above 6PP concentrations.

### 2.3 Seedling emergence and development

Seeds were sown into germination trays containing individual 52 cm^3^ cells. Each cell was filled with Lincoln University Horticulture Nursery 3–4-month standard potting mix (Potting mix composition: 80% composted bark, 20% pumice (grade 1-7), 3kg/m^3^ Osmocote^®^ Extract, 1 kg/m^3^ horticulture lime and 1kg/m^3^ Hydraflo). After filling, the trays were watered slowly and uniformly until maximum water-holding capacity (MWHC) was reached, defined as the point at which the substrate became fully saturated and free drainage was first observed from the bottom of the cells, indicating that no further water could be retained against gravity. The seeds were then sown at a depth of 1.5 cm. The trays were placed in a plant growth chamber at 25 °C ±2 °C, with a light cycle of 16 h light, 8 h dark, and 55% relative humidity. Fifteen seeds were sown per germination tray with three replicate trays per treatment arranged in a randomized block design.

**Figure 1:**
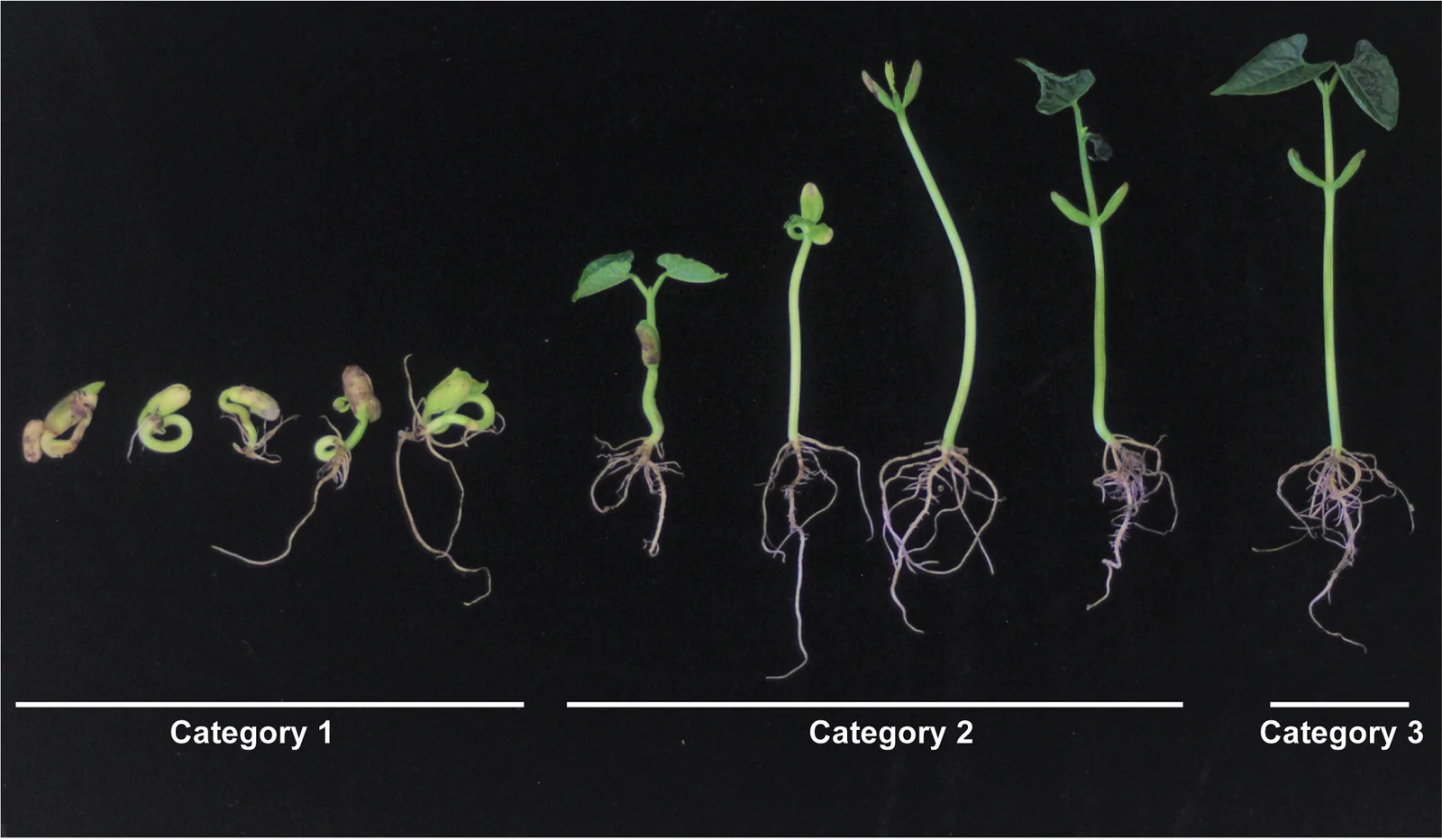
Classification of dwarf bean seedlings based on morphological development. Representative seedlings are shown across three categories (left to right). Category 1: seedling had emerged but showed abnormal development (e.g., visible shoot but poorly developed or abnormal development; curled shoot, no unifoliolate leaf, or roots appearing above surface). Category 2: seedling emerged with an apparently healthy but short shoot compared to a control seedling, one or both unifoliolate leaves developed atypically, or the seedling had only one unifoliolate leaf. Category 3: seedling showed normal shoot development, the shoot was completely straight, and the two unifoliolate leaves were the same size and unblemished.

Seven days after sowing, the emerged seedlings were counted, and the emergence percentage was calculated. Any seed that did not produce an emerged seedling was carefully removed from the potting mix and examined to determine if it had either (i) germinated physiologically (radicle emerged from the seed coat) but had not emerged from the soil, or (ii) died. The emerged seedlings were then visually examined and placed into one of three categories as described in **Figure 1**.

### 2.4 Drought stress experiment

#### 2.4.1 Experimental design

**Figure 2:**
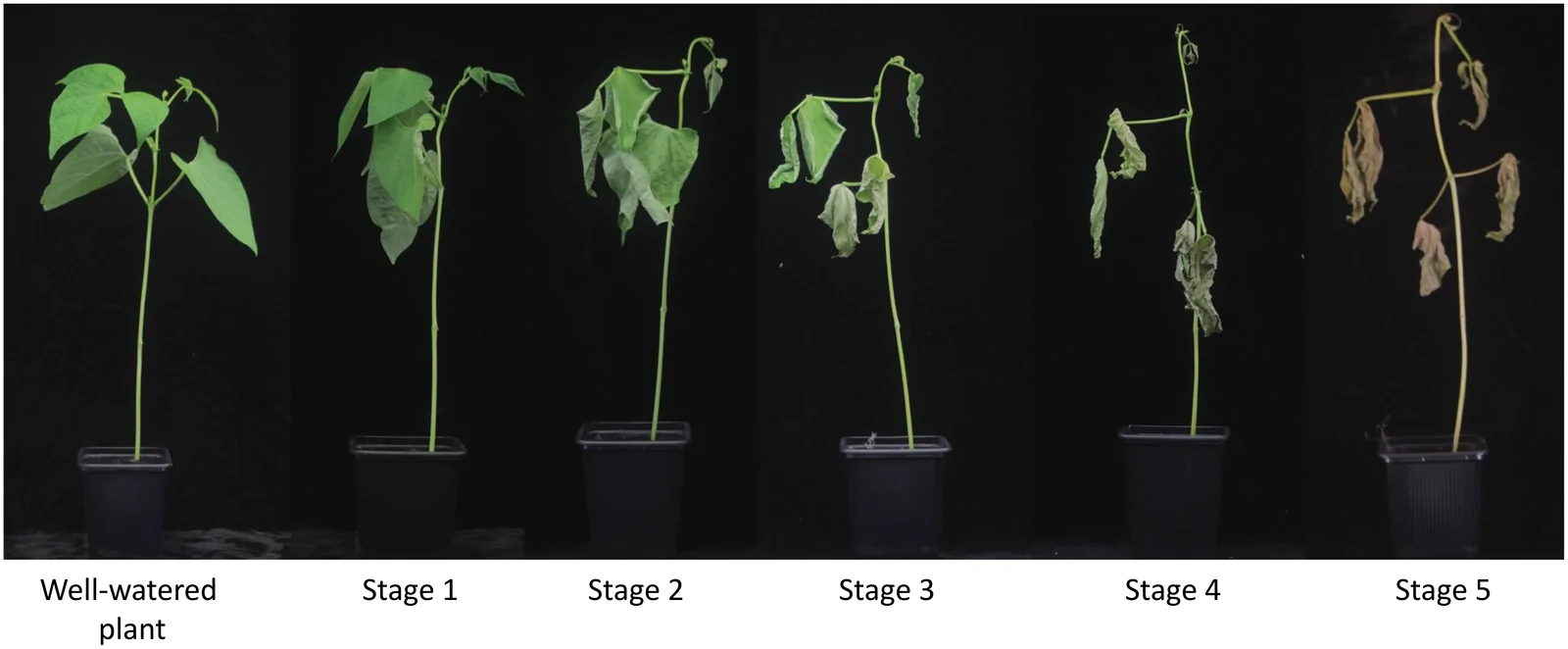
Visual scale of drought-induced wilting in dwarf bean plants. Representative plants are shown from a well-watered control (left) through five progressive stages of wilting (Stages 1–5), ranging from slight leaf drooping to complete desiccation. This scale was used to assess the severity of drought stress.

Based on the initial observation of plant response to 6PP seed treatment (see 2.3), the number of 6PP concentrations was reduced to only the three highest for the drought experiment. For each of the now four seed treatments, viz. polymer only and polymer plus 75 mM, 144 mM and 228 mM 6PP, a further set of seeds was sown in germination trays (15 seeds per tray x 3 replicate trays per treatment) using the method described above (see 2.3). The trays were arranged in a randomized block design, and after emergence, the plants were watered every second day by filling the saucers. Drought stress was applied 14 days after seedling emergence, by which time the seedlings had developed the first set of trifoliate leaves. The water supply was stopped, and the drought stress was continued for the five days following the appearance of stage 2 wilting symptoms (**Figure 2**). To prevent plant death during the drought treatment, the pots were maintained at 10% soil water content (SWC), as described by (Voroney et al., 2008). At day 22 after emergence, watering was resumed, and the plants were then left for a further 10 days to recover from the effects of the drought stress.

The wilting stage was assessed using a 1 to 5 scale (**Figure 2**): Stage 1(slightly wilted): leaves still green but with a slight downward angle. Stage 2 (moderately wilted): leaves tilted (less than 45 degrees), and leaf blades have started to curl. Stage 3 (severely wilted): leaves still green, the majority are at or near a 90° angle from the horizontal, and extensive leaf curling is noticeable; small necrotic zones are seen around the leaf margins. Stage 4 (nearly dead): most leaves exhibit severe necrosis and considerable leaf curling, with a predominant leaf angle of 90 degrees from the horizontal. Stage 5 (dead): necrosis on every leaf, severe curling, and brittle leaf blades.

#### 2.4.2 Data collection

##### 2.4.2.1 Measurement of plant growth-related parameters

Ten days after the water supply was restored, fifteen randomly selected plants per treatment were measured for plant growth-related parameters. Plants were carefully removed from the potting mix, rinsed with running water to remove any substrate particles, then blotted dry with filter paper to remove excess moisture. Root length was then measured with a ruler. The plants were then placed in a drying oven at 60 °C for 5 days, after which the dry weight was measured.

##### 2.4.2.2 Relative chlorophyll content

A SPAD (Soil Plant Analysis Development) chlorophyll meter (Konica Minolta, Japan) was used to measure relative chlorophyll content. The SPAD values were measured from the second fully developed trifoliate leaf from the top of the plant. Readings were taken from three sections of the leaf: i) near the leaf’s base, (ii) halfway between the leaf’s base and the tip, and (iii) near the tip of the leaf (**Supplementary Figure 1**). The average of the three data points was calculated. During the experiment, chlorophyll relative content was measured at three time points. 1) On day 14 after seedling emergence, just before the drought stress was initiated (pre-drought stress). 2) On plants undergoing drought stress (day 5 of the drought stress following the appearance of stage 2 wilting symptoms). 3) Ten days after the water supply was restored and the plants had recovered from the drought stress (post-drought stress). The plants were considered recovered when their leaves returned to turgidity.

##### 2.4.2.3 Gas exchange measurements (photosynthetic rate and stomatal conductance)

Gas exchange measurements, including photosynthetic rate and stomatal conductance, were conducted pre-, during, and post-drought stress using a LI-COR 6800 portable photosynthesis system (LI-COR Biosciences, Lincoln, NE, U.S.A.). Measurements were taken from fully expanded leaves of five randomly selected plants per treatment. As the plants were grown under controlled environmental conditions, all measurements were taken between eight and ten hours into the light cycle at a constant temperature of 25 °C, within a two-hour window.

##### 2.4.2.4 Leaf area

Two sets of trifoliate leaves (the first and second fully developed trifoliate leaves from the top of the plant) were cut from fifteen remaining plants for each treatment. The trifoliate leaves were then photographed individually and uploaded to Easy-Leaf Area software (Easlon and Bloom, 2014). To calculate the area of the leaf, one trifoliate leaf was placed on a white background along with a 4 cm^2^ red square, which provided a scale reference for the software to take the measurement (**Supplementary Figure 2**). Once the picture was taken and uploaded to the software, the color scale bar sliders were adjusted to identify the leaf and the scale reference. The background was removed, and the measurements were taken (**Supplementary Figure 2**) (Easlon and Bloom, 2014).

### 2.5 Systemic transcriptional changes in plants treated with 6PP under well-watered and drought stress conditions

#### 2.5.1 Plant growth and drought stress experiment

Templeton silt loam soil was collected from an organic vineyard at Lincoln University, New Zealand. The soil was sieved through a 0.5 cm mesh and mixed with perlite at a 3:1 ratio (soil: perlite). Plastic pots (1.5 L; 14 cm height × 14 cm diameter) were disinfected with sodium hypochlorite (500 ppm), rinsed thoroughly to eliminate any trace of sodium hypochlorite, and then filled with 2.7 kg of the soil/perlite mixture. Pots were saturated with water to reach their maximum water-holding capacity, and after 24 hours, saucers containing water were placed beneath each pot to maintain soil moisture. Three seeds were sown per pot at a depth of 1.5 cm. Seedlings were thinned to one uniform, healthy plant per pot 5–7 days after emergence.

The primary aim of the experiment was to assess the transcriptional profiles of plants treated with 6PP and the untreated control under both well-watered and drought stress conditions. The experiment was conducted in a Conviron BDW120 plant growth chamber at the Biotron Complex, Lincoln University, New Zealand. The chamber allowed precise regulation of growth conditions, including relative humidity, light, and temperature.

Environmental settings within the plant growth chamber were as follows: the photoperiod was set to 16 h light: 8 h dark, using a combination of incandescent and fluorescent lights with a wavelength range of 400–700 nm and a light intensity of 400 µmol m^-^² s^-^¹. Daytime temperature was maintained at 25 ± 2 °C, and at night it was reduced to 20 ± 2 °C to mimic natural temperature fluctuations. Relative humidity was set at 50%-60% throughout the experiment.

Ten plastic trays (48 cm × 34 cm × 5 cm) were placed in each of two Biotron chambers. Each chamber contained five trays per treatment, with each tray holding five pots. The experimental treatments were as follows: (1) Control treatment: seeds coated with polymer only, and (2) 6PP treatment: seeds coated with 114 mM 6PP combined with polymer. The plants were watered as required by adding water to the saucer of each pot every 2 days and left to grow until they developed their second trifoliate leaf. Once this developmental stage was reached, the drought stress was imposed by suspending irrigation for 16 days until stage 2 wilting symptoms were observed (**Figure 2**).

When drought-stressed plants reached wilting stage 3, three replicates of a composite of one set of trifoliate leaves from five randomly selected plants per treatment were collected. Each leaflet was detached from the petiolule, immediately frozen in liquid nitrogen and ground into a fine powder using a sterile mortar and pestle. During the grinding process, liquid nitrogen was added as required to keep the plant material frozen and avoid RNA degradation. The resulting plant material was transferred to a 50 mL Falcon tube and stored at -80 °C until RNA isolation.

#### 2.5.2 Total RNA isolation and purification

Total RNA was extracted from 100 mg of ground plant material using TRIzol reagent (Invitrogen, U.S.A). Samples were homogenized in 1 mL TRIzol by vortexing, then 200 µL chloroform was added, incubated at room temperature for 3 minutes, and centrifuged (12,000 rpm, 4 °C, 15 min). The aqueous phase was transferred to a new tube, mixed with 500 µL isopropanol, incubated on ice for 10 minutes, and centrifuged (12,000 rpm, 4 °C, 10 min). The total RNA pellet was washed with 75% ethanol, centrifuged (12,000 rpm, 4 °C, 5 min), air-dried, and resuspended in 35 µL DEPC-treated water, then incubated at 55 °C for 10 minutes.

Total RNA was subsequently purified using a modified Plant Total RNA Extraction Miniprep System (VIOGENE, U.S.A.). Briefly, 35 µL of total RNA solution, 450 µL of RX buffer, and 230 µL of 98% ethanol were added, mixed, and loaded onto a spin column. After centrifugation (10,000 rpm, 1 min), the flow-through was discarded. The column was washed with 500 µL WF buffer (14,000 rpm, 1 min), followed by two washes with 700 µL WS buffer. A final spin (14,000 rpm, 3 min) was performed before RNA was eluted with 50 µL RNase-free water and stored at −80 °C. RNA concentration and purity were measured with a NanoDrop spectrophotometer (NanoDrop, Technologies Inc.), and integrity was verified using an Agilent Bioanalyzer (Agilent Technologies, Palo Alto, Calif.). Only samples with RIN > 6.8 were used in downstream analyses.

#### 2.5.3 Illumina sequencing

The cDNA libraries were prepared by Novogene (HK) Co., Ltd, and sequenced on an Illumina^®^ NovaSeq™ 6000 (Modi et al., 2021). The samples were sequenced with 150 paired-end reads.

##### 2.5.3.1 Analysis of read data

Raw reads were trimmed, and quality control was performed with CLC Genomics Workbench 21.0.4 (QIAGEN), where parameters such as read length, base quality, nucleotide composition, and contamination were assessed. High-quality reads were aligned to the *Phaseolus vulgaris* BAT93 reference genome (Genome ID 20365, CoGe platform, https://www.ncbi.nlm.nih.gov/datasets/genome/GCA_040616515.1/) using default parameters (mismatch cost = 2; insertion/deletion cost = 3; length fraction = 0.8; similarity fraction = 0.8; max hits per read = 10). Gene expression levels were normalized using the transcripts per million (TPM) method. Differential expression analysis was conducted using the CLC RNA-Seq Analysis Toolkit, and differentially expressed genes (DEGs) were identified based on a log_2_ fold change ≥ 0.6, false discovery rate (FDR) ≤ 0.01, and Bonferroni ≤ 0.05. Data visualizations, including volcano plots, principal component analysis (PCA), and Venn diagrams, were also generated within the CLC platform.

##### 2.5.3.2 Functional enrichment analysis

Functional enrichment analysis was performed using Cytoscape v3.9.1 (Shannon et al., 2003) and ShinyGO 0.82 (Ge et al., 2019). The analysis was initiated by constructing a network of DEGs using the StringApp v2.2.0 plugin in Cytoscape (Doncheva et al., 2019). Functional enrichment was then carried out in ShinyGO 0.82, and only statistically significant terms were retained across four annotation categories: Gene Ontology (GO) Biological Process, GO Molecular Function, GO Cellular Component, and KEGG pathways.

### 2.6 Statistical analysis

The statistical analysis of seedling emergence data was performed in R (RStudio version 2024.04.0) (Team, R, 2015) using a generalized linear model with a binomial distribution. For the data analysis of growth-related parameters, an ANOVA was used. Analyses were conducted using the GenStat 21st edition software (VSN International, UK). The individual data sets were compared using Tukey’s Honest Significant Difference (HSD) Test at the 5% significance level.

### 2.7 MAPK phosphorylation

MAPK phosphorylation was evaluated in leaf tissue from control and 6PP-treated plants grown under well-watered and drought conditions. Frozen tissue was homogenized for total protein extraction in buffer supplemented with 1 mM orthovanadate, phosphatase inhibitors, and protease inhibitor tablets. Protein samples (25 µg) were separated by SDS-PAGE, transferred to membranes, and immunodetected with antibodies against phosphorylated MAPKs and total MAPKs. Phosphorylated MAPKs were probed with a Rabbit anti ERK1 (pThr202/pTyr204)/ERK2 (pThr185/pTyr187) antibody (Cat No. AHP2608, Bio-Rad) following the manufacturer’s protocol. Western blot signals were acquired using the ChemiDoc MP Imaging System and quantified using quantification and using Image Lab software, version 6.0.1 (Bio-Rad). Three technical replicates were used for the quantification.

## 3 Results

### 3.1 Effect of 6PP-coating on seedling emergence and seedling development

The percentage of seedlings that emerged did not differ among the treatments, except for the 228 mM 6PP treatment, where 29% of the seeds failed to produce an emerged seedling (**Table 1**). This treatment was selected following a preliminary dose-ranging assay, which indicated that higher 6PP concentrations were required to produce a clear biological response under our experimental conditions. Of these, 8% of the seeds were dead, while the remaining 21% had germinated physiologically but failed to emerge. When the emerged seedlings were individually classified into three groups, 70-74% were placed in category 3 (normal seedlings) across all treatments except for 228 mM 6PP, which had only 58% in this category (**Table 1**), and this was significantly different from the other treatments. For categories 1 and 2, the 228 mM 6PP treatment had more seedlings than other treatments (**Table 1**). Compared with the polymer control, the other treatments either did not differ or showed minor differences in the three classification categories (**Table 1**).

**Table 1:** Effect of 6PP on dwarf bean seedling emergence after seven days in a controlled environment.

Treatment	% Seedling	% seedlings in each morphological category		
	emerged	Category 1	Category 2	Category 3
Polymer	93	10	19	71
polymer + 75 μM	92	9	18	73
polymer + 114 μM	90	10	16	74
polymer + 228 μM	92	12	18	70
polymer + 75 mM	93	13	16	71
polymer + 114 mM	89	14	16	70
polymer + 228 mM	71*	20	22	58**

Category 1: abnormal seedling development; Category 2: partially developed seedlings; Category 3 normal, healthy seedlings.

Significance *p <0.05; **p <0.01. The emerged seedling categories were assigned by visual assessment.

### 3.2 Plant growth-related parameters across different stages of drought stress

#### 3.2.1 Plant morphology under drought stress

**Figure 3:**
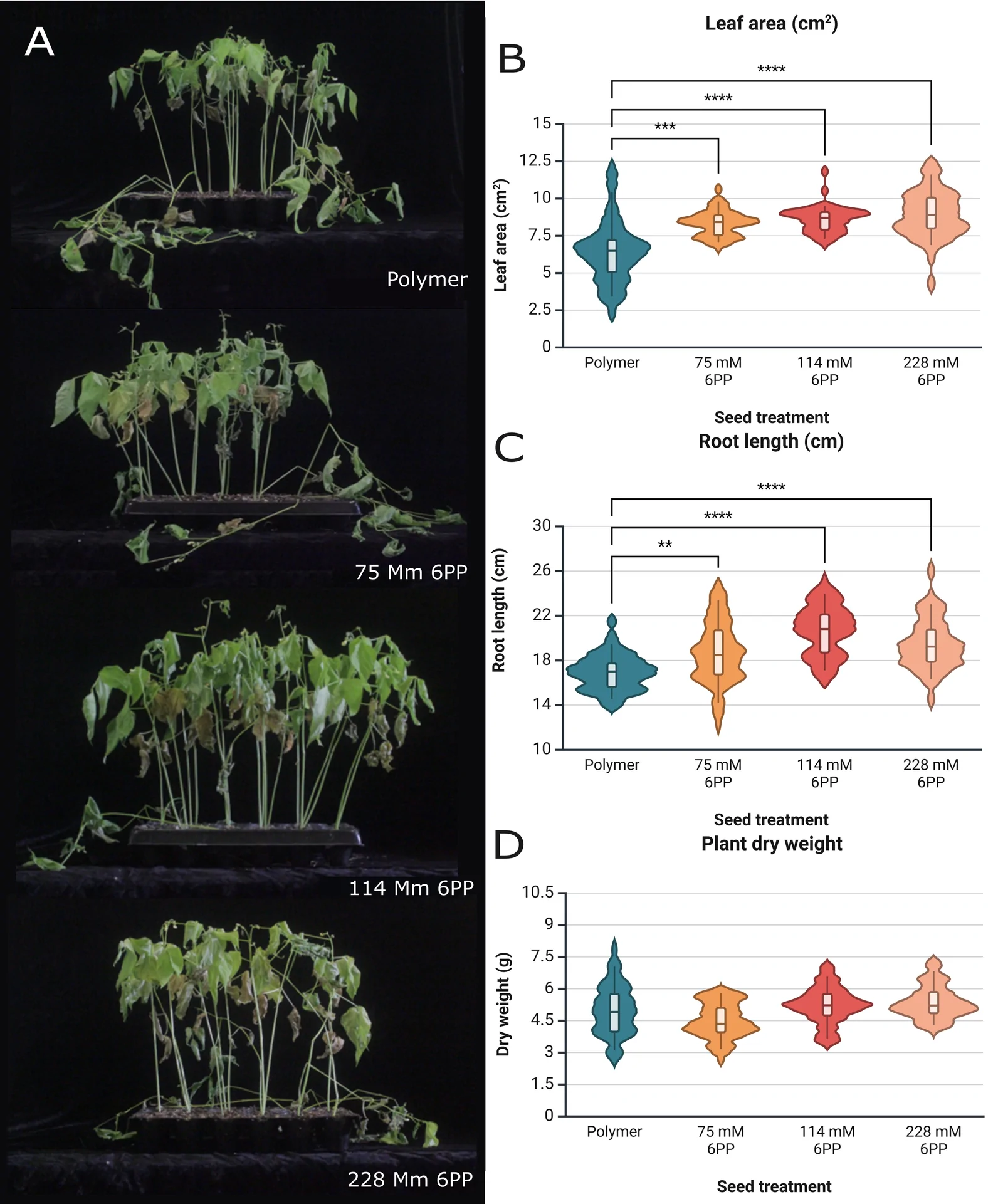
Influence of 6PP seed treatment on growth, morphology, and drought recovery in dwarf bean plants. **(A)**. Appearance of dwarf bean plants on day 8 of the drought stress.. **(B)** Leaf area per plant 10 days after the end of drought stress. **(C)**. Root length 10 days after the drought stress ended. **(D)**. Plant dry weight 10 days after the drought stress ended. For **(B–D)**, asterisks indicate statistically significant differences among treatments (*p < 0.05, **p < 0.01, ***p < 0.001, ****p < 0.0001) based on one-way ANOVA followed by Tukey’s Honest Significant Difference (HSD) test. Violin plots show the distribution of three replicates per treatment. Embedded box plots indicate medians and quartiles; whiskers extend to 1.5 × the interquartile range.

Plants grown from seeds coated with 6PP had a significantly greater leaf area (p < 0.05) compared to the polymer-only control. On the eighth day of drought stress, these plants displayed visibly greater vigor than the control (**Figure 3A**). Leaf area increased by 30%, 34%, and 40% in plants from seeds coated with 75 mM, 114 mM, and 228 mM 6PP treatments, respectively (**Figure 3B**).

Root length was also significantly enhanced in all 6PP seed-coating treatments (p < 0.05) (**Figure 3C**). Plants from seeds coated with 75 mM 6PP showed a 10% increase in root length after the recovery period, while the 114 mM treatment showed the greatest increase of 22%. The 228 mM treatment improved root length by 15%.

Plant dry weight, which ranged from 4.95g for the polymer-only control to 5.36g in plants from seeds coated with 228 mM 6PP, did not differ significantly among the treatments (**Figure 3D**).

#### 3.2.2 Chlorophyll relative content

**Figure 4:**
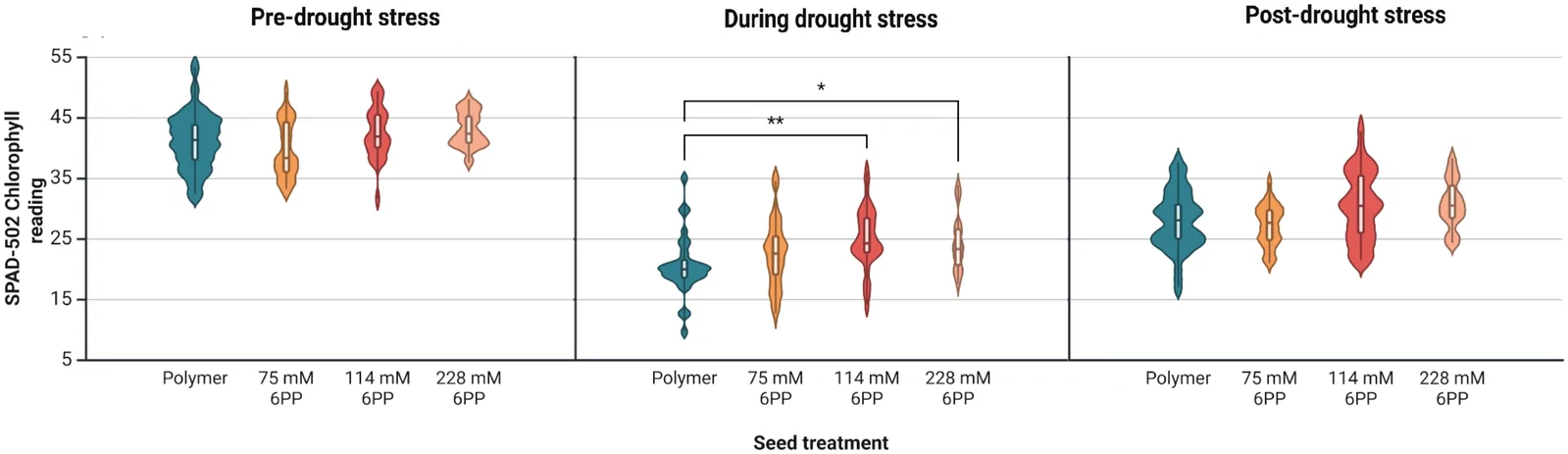
Effect of 6PP seed coating on relative chlorophyll content in dwarf bean leaves measured before, during, and after drought stress. Violin plots show the distribution of three replicates per treatment. Asterisks indicate statistically significant differences among treatments (*p < 0.05, **p < 0.01), based on one-way ANOVA followed by Tukey’s Honest Significant Difference (HSD) test. Violin plots display the distribution of three replicates per treatment. The horizontal line represents the median, the bar indicates the interquartile range, and the vertical line represents the data range.

Before drought stress, SPAD readings for plants across all 6 PP-coated seed treatments did not differ from the polymer-only control (**Figure 4**). On the eighth day of drought stress, plants from seeds coated with 114 mM and 228 mM showed significantly higher chlorophyll content than the control (p< 0.05), with increases of 22% and 19%, respectively (**Figure 4**). Post-drought stress SPAD readings of plants from 6PP-coated seeds did not differ significantly from the control.

#### 3.2.3 Gas exchange response pre-, during and post-drought stress

**Figure 5:**
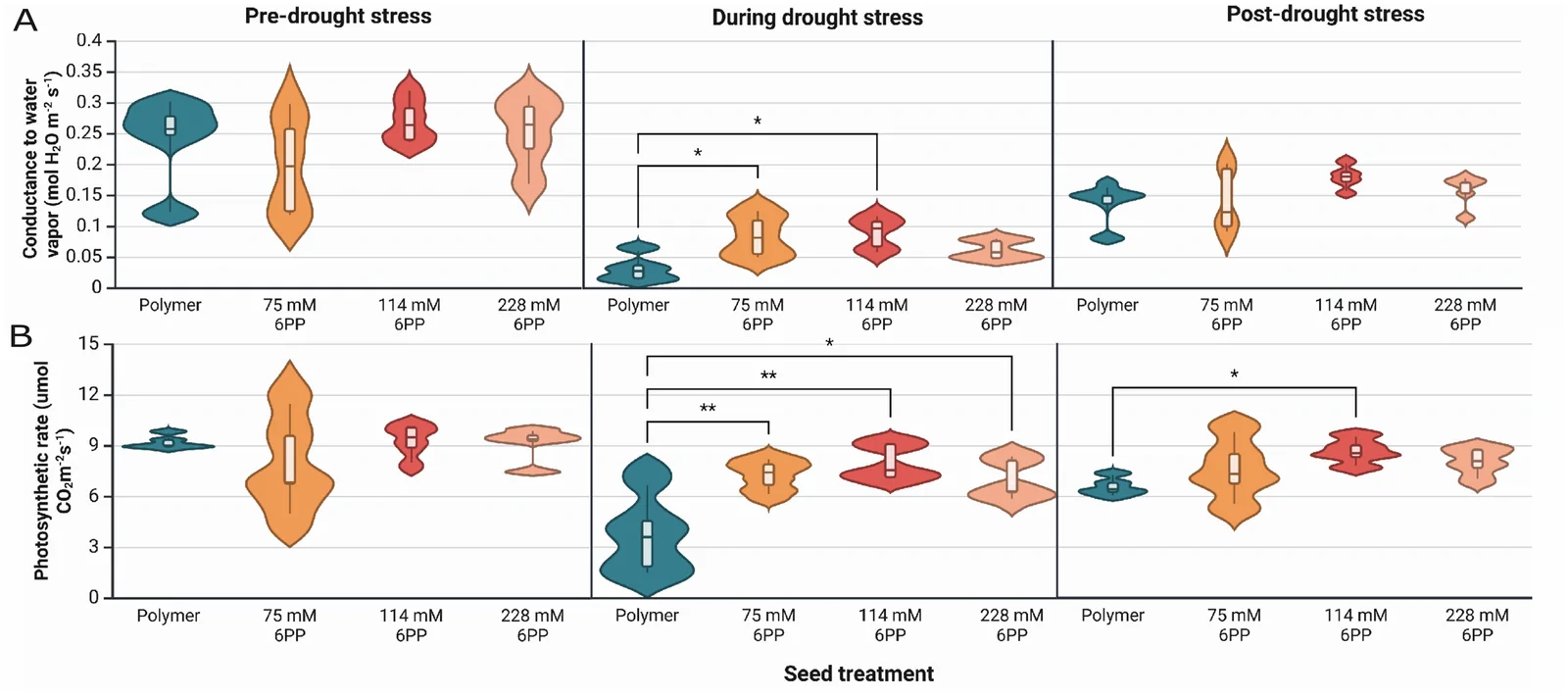
Effect of 6PP seed coating on dwarf bean leaf gas-exchange parameters measured before, during, and after drought stress. **(A)** stomatal conductance and **(B)** photosynthetic rate. Violin plots show the distribution of three replicates per treatment. Embedded box plots indicate the median and quartiles, and whiskers extend to 1.5 times the interquartile range. Asterisks indicate statistically significant differences among treatments (*p < 0.05, **p < 0.01) based on one-way ANOVA followed by Tukey’s Honest Significant Difference (HSD) test.

Before the drought stress was applied, no significant differences were observed in either photosynthetic rate or stomatal conductance among treatments (**Figures 5A, B**).

During drought stress, stomatal conductance was enhanced by 171% (75 mM 6PP), 186% (114 mM 6PP), and 98% (228 mM 6PP) relative to the polymer control (**Figure 5A**). However, these differences were statistically significant only for the 75 mM and 114 mM 6PP treatments (p < 0.05 and p < 0.01, respectively). Similarly, the photosynthetic rate in plants treated with 6PP was significantly higher than in the polymer control plants, with increases of 95% (75 mM 6PP), 115% (114 mM 6PP), and 88% (228 mM 6PP) (**Figure 5B**). Among all treatments, the 114 mM 6PP rate consistently showed the highest responses for both parameters (**Figures 5A, B**).

After drought stress, partial recovery was observed across all treatments. There were no differences in stomatal conductance (**Figure 5A**) but for photosynthetic rate the 75 mM 6PP treatment had a significantly higher rate than the polymer control (p *<* 0.05) (**Figure 5B**).

### 3.3 Transcriptional response to 6PP seed coating in drought protection

Under well-watered conditions, 6PP seed treatment elicited a limited but distinct transcriptional response, with 49 genes differentially regulated (**Supplementary Table 1**). The response was dominated by genes linked to ribosomal function and photosynthesis, suggesting effects on protein synthesis and chloroplast activity even in the absence of drought stress. Importantly, one of the most differentially regulated genes was involved in abscisic acid metabolism (PHAVU_002G122200g), suggesting a potential role for 6PP in priming ABA-associated pathways. The dataset also included stress- and development-related transcripts, such as ABA 8’-hydroxylase, senescence-associated protein, exordium-like proteins, and a proline-rich cell wall protein, together with a form in-like protein and urease accessory protein. Overall, these results suggest coordinated transcriptional changes affecting photosynthesis, protein synthesis, and stress adaptation.

To assess whether early exposure to 6PP via seed coating modifies the molecular response of plants to water deficit, we performed a comparative transcriptomic analysis. Plants grown from seeds treated with polymer-only (control) were compared with those coated with polymer containing 114 mM 6PP. This approach allowed us to determine whether 6PP pre-treatment induced transcriptional modifications during drought stress, consistent with a stress-priming effect.

**Figure 6:**
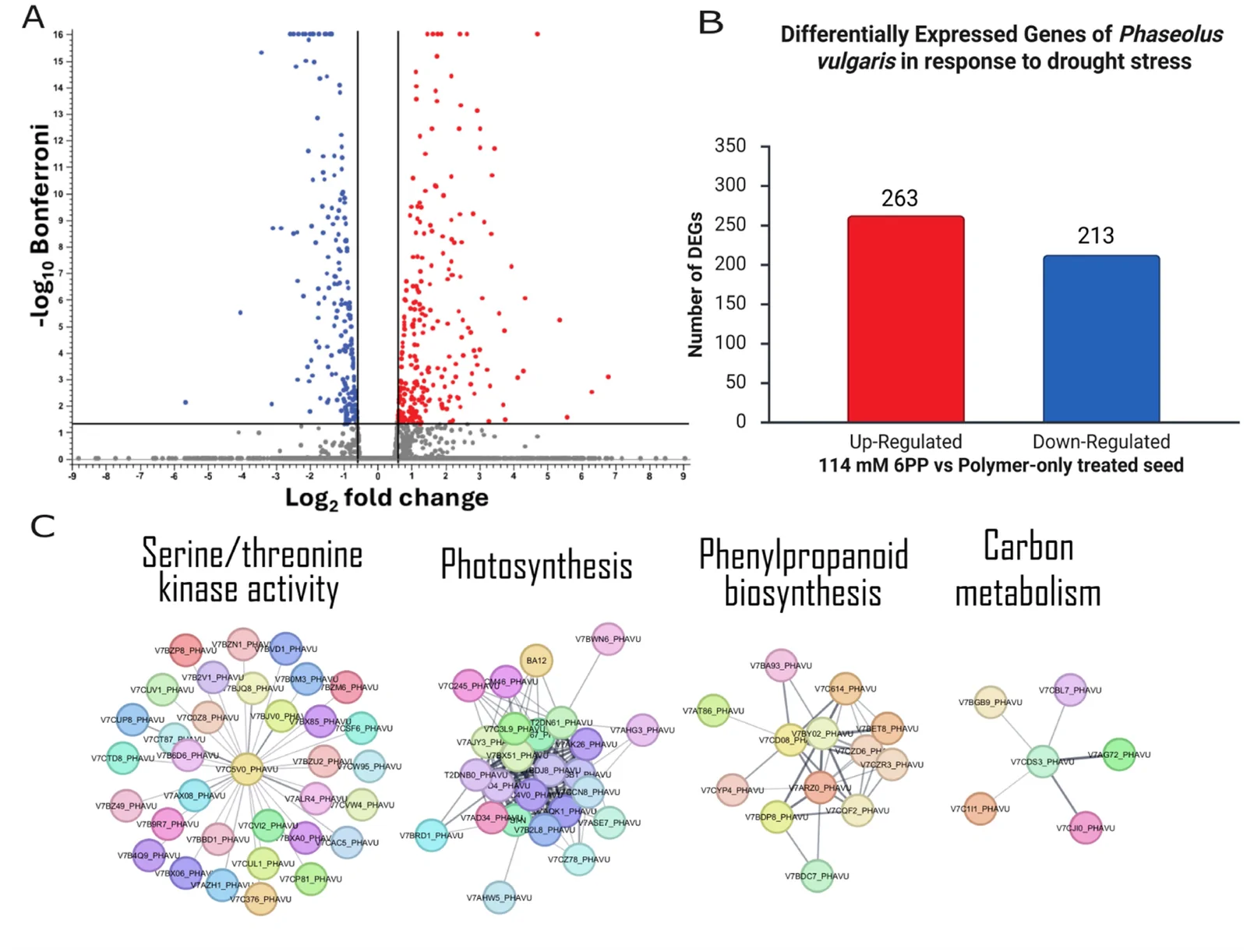
Differential gene expression and functional network analysis of dwarf bean to drought stress following 6PP seed coating. **(A)** Volcano plot of differentially expressed genes (DEGs). Red (up-regulated) and blue (down-regulated) dots represent DEGs chosen under the following criteria: log_2_ fold change ±0.6, false discovery rate (FDR) value ≤ 0.01, and Bonferroni value ≤ 0.05. **(B)** Regulation trend bar chart with the number of up-regulated (red) and down-regulated (blue) DEGs. **(C)** Main functional STRING protein-protein interaction networks of drought-stressed dwarf bean. Networks were visualized using Cytoscape.

A volcano plot was generated to visualize the distribution of differentially expressed genes (DEGs) between plants grown from 6PP-coated seeds and those from the polymer-only control (**Figure 6A**). The analysis identified 476 DEGs (**Supplementary Table 2**), including 263 upregulated and 213 downregulated genes (**Figure 6B**). These results indicate that 6PP, when applied as a seed coating, induces distinct transcriptional reprogramming in plants subjected to drought stress.

All the DEGs (476 genes) (**Figure 6B**), identified in response to 6PP, were analyzed using the STRING database to assess protein–protein interactions (PPI) and functional enrichment. The analysis revealed 249 nodes and 391 edges (**Supplementary Table 3**), indicating a well-connected interaction network. The proteins encoded by these DEGs were primarily associated with kinase activity (signaling), phenylpropanoid biosynthesis, and the photosynthetic process (**Figure 6C**).

**Figure 7:**
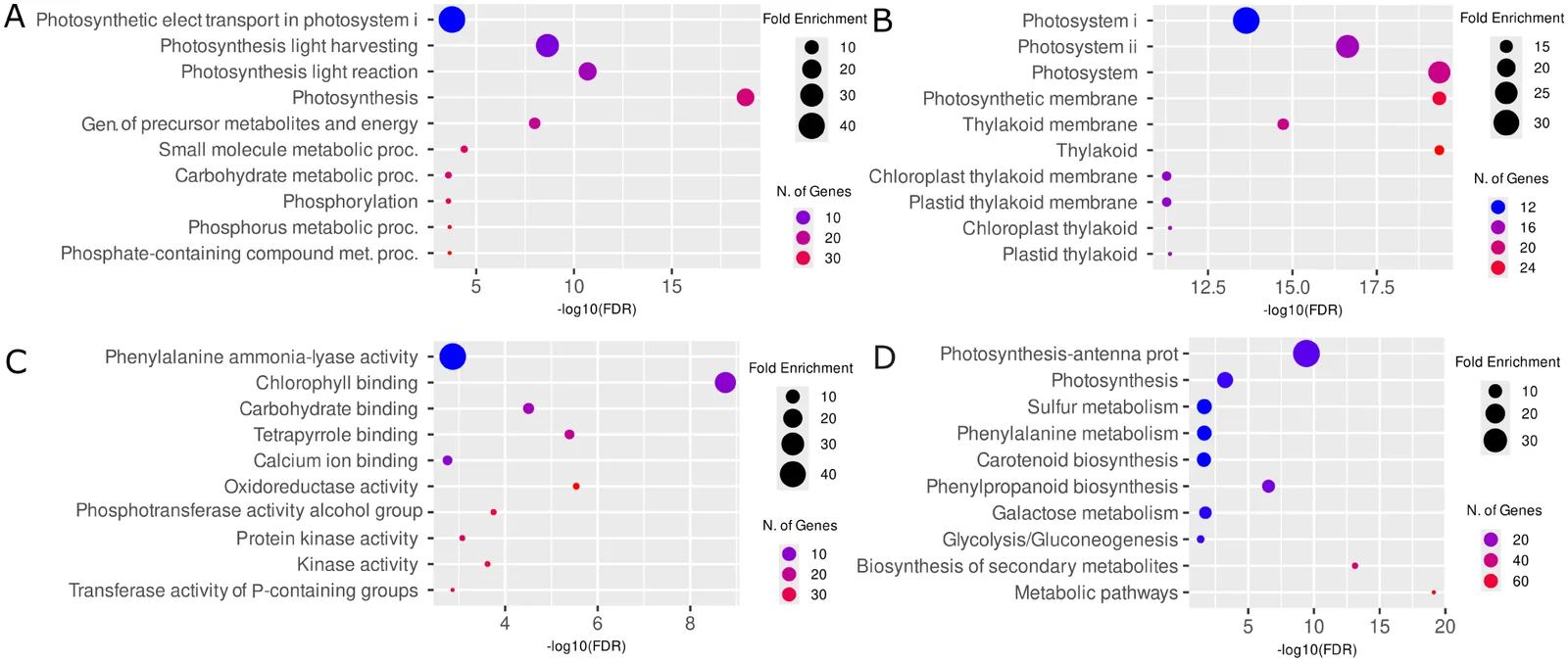
Gene Ontology (GO) and KEGG pathway enrichment analysis of differentially expressed genes (DEGs) in drought-stressed dwarf bean plants grown from 6PP-treated seeds. Dot plots show fold enrichment, significance (–log_10_ FDR), and the number of genes associated with each GO term or KEGG pathway. **(A)** Biological Process, **(B)** Cellular Component, **(C)** Molecular Function, and **(D)** KEGG pathways. GO analysis was performed using ShinyGO v0.82, and only statistically significant terms were retained.

The 476 DEGs identified in drought-stressed plants grown from 6PP-treated seeds were examined using GO functional enrichment and KEGG pathway analyses (**Figure 7**). The dot plot displays fold enrichment, the significance level (–log_10_ FDR), and the number of genes associated with each category or pathway. The enrichment analysis revealed an activation of processes associated with plant defense, photosynthesis, and signal transduction.

Within the biological process category, the functional analysis showed a significant enrichment of pathways related to protein phosphorylation, precursor generation and carbohydrate metabolic processes, and photosynthetic related processes (**Figure 7A**). These categories suggest an adjustment of metabolic and defense mechanisms in response to drought stress. The enrichment in photosynthetic electron transport, generation of precursor metabolites and energy, and carbohydrate metabolic processes, suggests an enhanced accumulation of precursors for the synthesis of secondary metabolites. The enrichment of photosynthesis-related processes, including the photosynthetic electron transport chain and light-harvesting categories, indicates that 6PP treatment helped maintain a greater photosynthetic performance during drought stress.

The cellular component category showed strongly enriched categories associated with photosynthetic structures, such as the chloroplast envelope, thylakoids, and photosystems I and II (**Figure 7B**). This enrichment reinforces the idea that 6PP treatment supports the integrity and activity of photosynthetic organelles under drought stress conditions. The enrichment in these categories is consistent with physiological measurements showing higher chlorophyll content and photosynthetic rate in 6PP-treated plants during drought stress.

The molecular function category highlighted enriched activities in signal transduction and energy production, including protein kinase activity, phosphotransferase activity, and calcium ion binding. All these categories play essential roles in phosphorylation-mediated signaling and cellular communication during stress (**Figure 7C**). The presence of kinase-related terms suggests the activation of signaling pathways. In contrast, the enrichment of oxidoreductase and chlorophyll-binding activities indicates enhanced redox regulation and pigment stabilization under stress conditions compared with control plants. The latter functions are closely associated with photosynthetic processes.

Finally, an enrichment in the KEGG pathway related to metabolic routes associated with plant defense and photosynthetic processes was identified (**Figure 7D**). The most enriched pathways were phenylpropanoid and flavonoid biosynthesis, oxidoreductase activity, and photosynthesis, supporting the hypothesis that 6PP seed coating stimulated the production of protective secondary metabolites, such as lignin and flavonoids, which can strengthen cell walls and reduce oxidative stress. This suggests a link between enrichment of the phenylpropanoid pathway and primary energy production.

The significantly enriched GO categories and KEGG pathways highlight the main functional domains affected by 6PP treatment under drought stress. These include biological processes related to photosynthesis, phosphorylation, and carbohydrate metabolism; cellular components associated with photosynthetic membranes and thylakoid structures, and molecular functions involving kinase, oxidoreductase, and chlorophyll-binding activities (**Table 2**).

**Table 2:** Summary of significantly enriched Gene Ontology (GO) categories and KEGG pathways in drought-stressed plants.

GO category	Term name	Description	FDR value	# genes
**GO Biological Process**	GO:0009698	Phenylpropanoid metabolic process	2.57E-06	10
**GO Biological Process**	GO:0022900	Electron transport chain	1.60E-04	9
**GO Biological Process**	GO:0098542	Defence response to organisms	0.002	14
**GO Biological Process**	GO:0009807	Lignan biosynthetic process	0.0028	3
**GO Biological Process**	GO:0009867	Jasmonic acid mediated signalling pathway	0.0049	5
**GO Biological Process**	GO:0009767	Photosynthetic electron transport chain	0.0051	4
**GO Cellular Component**	GO:0009521	Photosystem	8.55E-22	18
**GO Cellular Component**	GO:0098807	Chloroplast thylakoid membrane protein	1.01E-05	4
**GO Cellular Component**	GO:0009941	Chloroplast envelope	5.40E-04	10
**GO Cellular Component**	GO:0009654	Photosystem II oxygen evolving complex	0.0041	3
**GO Molecular Function**	GO:0046906	Tetrapyrrole binding	5.75E-11	19
**GO Molecular Function**	GO:0016491	Oxidoreductase activity	9.13E-10	32
**GO Molecular Function**	GO:0045548	Phenylalanine ammonia-lyase activity	0.0027	3
**GO Molecular Function**	GO:0004097	Catechol oxidase activity	0.027	2
**KEGG Pathways**	pvu00940	Phenylpropanoid biosynthesis	4.32E-08	11
**KEGG Pathways**	pvu00195	Photosynthesis	1.11E-07	7
**KEGG Pathways**	pvu00941	Flavonoid biosynthesis	1.30E-04	5

The table presents the GO category, term name, description, False Discovery Rate (FDR) value, and the number of genes associated with each term. The table highlights enriched terms in three GO categories: Biological Process, Cellular Component, and Molecular Function, as well as significant KEGG pathways.

**Figure 8:**
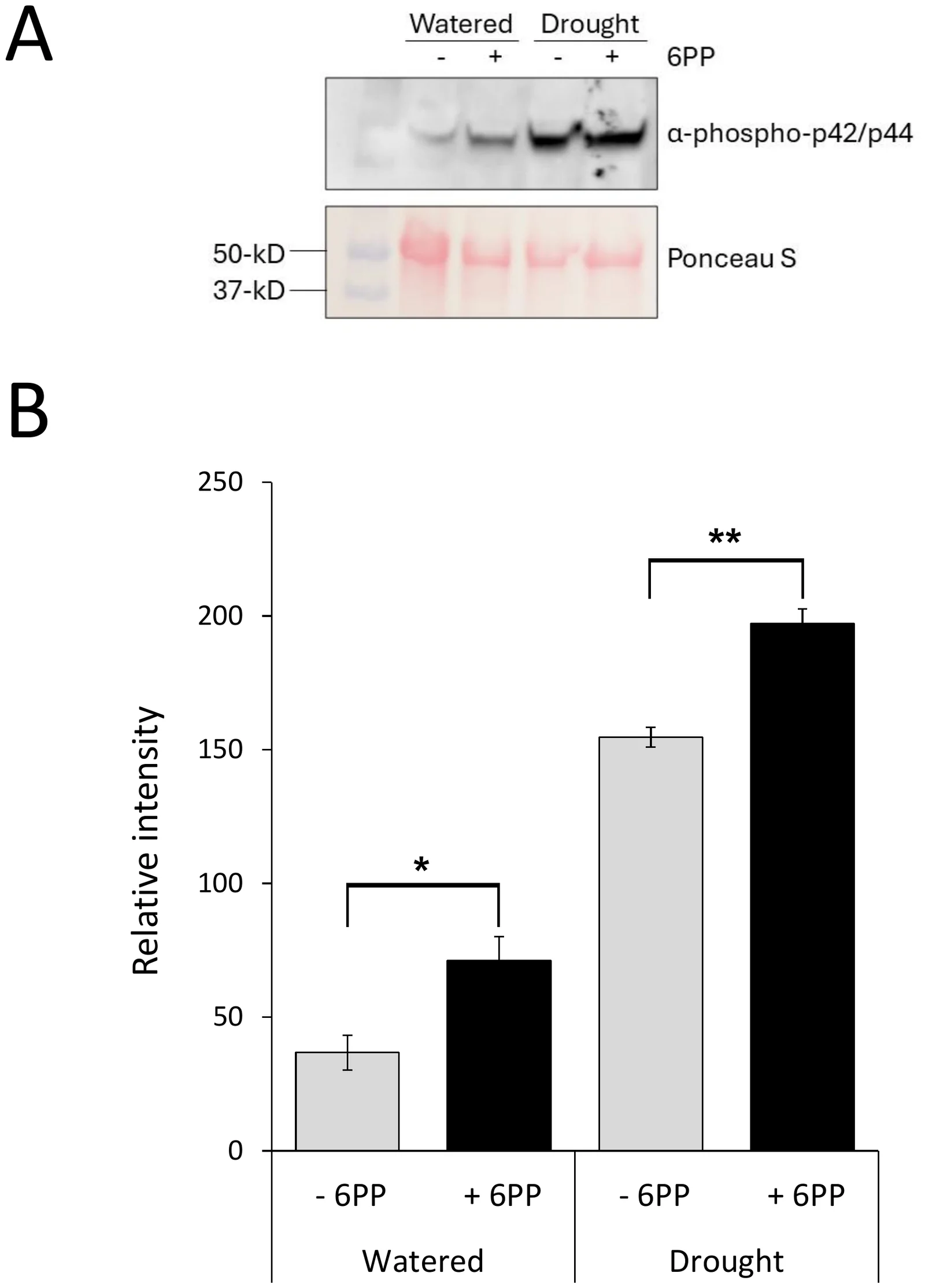
Immunoblot analysis of phosphorylated MAPKs in leaves of *Phaseolus vulgaris* grown from polymer-coated control seeds (−) or seeds coated with polymer containing 6PP (+) under well-watered and drought-stressed conditions. **(A)** Total protein extracts were prepared from leaf tissue, and 15 μg of total protein per sample was separated by SDS-PAGE and transferred to a PVDF membrane. Phosphorylated MAPKs were detected using an anti-phospho-p42/44 MAPK (ERK1/2) antibody. Ponceau S staining is shown as a loading control to confirm equal protein loading between samples. **(B)** Relative signal intensity was quantified from two independent blots prepared from the same samples used for transcriptome analysis. Data are shown as mean ± SD. Statistical significance is indicated as *p<0.0244; p<0.0244 and **p<0.0015.

To determine whether the enrichment of phosphorylation-related pathways identified by transcriptomic analysis corresponded to protein-level activation of signaling, MAPK phosphorylation was examined in plants grown under well-watered and drought conditions (**Figure 8**). MAPK phosphorylation was detected under both conditions, but the signal was markedly stronger in drought-stressed plants, with a significant increase in the samples treated with 6PP compared with the corresponding polymer controls (**Figure 8**). These results indicate that 6PP enhances MAPK phosphorylation and activation, particularly under drought stress, providing protein-level evidence that supports the transcriptomic enrichment of phosphorylation-mediated signaling pathways.

## 4 Discussion

This study investigated whether the volatile organic compound 6PP, applied via seed coating, influences seedling emergence, plant development, and molecular reprogramming (priming) that enhances drought tolerance in the dwarf bean cv. Messi. *Trichoderma* has been previously reported to enhance plant drought tolerance (Bae et al., 2009; He et al., 2022) but the molecules underlying this tolerance have not been identified. The only previous report of coating seeds with a polymer in combination with 6PP is that of Carillo et al. (2020), who evaluated the effect of 6PP on plum tomato fruit quality and the possible modulation of plant metabolism under ideal growth conditions. They found no significant differences in tomato fruit number, fruit weight, or plant yield compared to the control. Interestingly, there was a significant increase of 52% in lycopene content when compared to that of the untreated seeds, suggesting that while 6PP can promote plant growth under optimal concentrations (El-Hasan and Buchenauer, 2009; Garnica-Vergara et al., 2016; Lee et al., 2016; Pascale et al., 2017). It can also enhance plant immune responses by inducing defense-associated enzyme production, such as peroxidases, polyphenol oxidases, and glucanases (Carillo et al., 2020; El-Hasan and Buchenauer, 2009; Kottb et al., 2015). Lycopene, besides its antioxidant properties, is also an essential pigment in photosynthesis, allowing the plant to expand the range of light wavelengths usable for photosynthetic reactions (Bashan et al., 2006). The present study, therefore, aimed to assess whether 6PP can induce the production of defense-related molecules through plant transcriptional reprogramming, particularly in drought-stressed plants.

Previously published results have highlighted a concentration-dependent effect that 6PP exerts on plants. For instance, when 6PP was added to Murashige & Skoog growing medium in concentrations ranging from 120-280 µM, the emergence of maize was reduced by 40% to 60% (El-Hasan and Buchenauer, 2009). In wheat, when 10 mM of 6PP was added to the potting mix, the 6PP metabolite significantly inhibited coleoptile growth compared with the control (Cutler et al., 1986). Maize seedling growth was retarded when maize seeds were germinated in a medium containing 0.06-1.8 mM of 6PP. In contrast, when maize seedlings were treated with 1.2 mM 6PP using drenching, plant growth was promoted. A similar growth-promoting effect was observed when tomato leaves were sprayed with 10 µM of 6PP (Cutler et al., 1986; Degani et al., 2021; El-Hasan and Buchenauer, 2009; Vinale et al., 2008). The interest in identifying the highest concentration of 6PP that can be applied via seed coating was to avoid the reported negative effects (low germination percentage, abnormal seedling emergence, growth retardation) that 6PP can induce in plants when applied at high concentrations. Most commercially available polymers are designed to protect microbes rather than to stabilize bioactive compounds (Sun et al., 2023). Therefore, the optimal approach was to apply the maximum concentration of 6PP that did not cause deleterious effects at any developmental stage, ensuring both efficient delivery and biological activity.

### 4.1 Effect of 6PP-coating on seedling emergence and seedling fitness

The percentage of non-emerged seedlings from the 228 mM 6PP seed treatment was almost three times higher than the rest of the treatments assessed. This failure to emerge was mostly due to seedling abnormalities that prevented them from breaking through the soil surface. One of the most common abnormalities was the absence of a shoot, or the production of an abnormal shoot, e.g., a curled-up shoot, sometimes with the meristem pointing into the soil, or the roots pushed out of the soil because the shoot was pushing against the substrate. Seeds which had failed to germinate were dead (El-Hasan and Buchenauer, 2009). reported a negative effect of 6PP on emergence and elongation of maize when seeds were germinated on filter paper impregnated with 1.2 mM or 1.8 mM of 6PP. Although the delivery method of the molecule differed from that used in the present research, the negative effects reported on seedling emergence are consistent with these results. Garnica-Vergara et al. (2016) tested the effect of micromolar concentrations of 6PP added to 0.2X MS medium on biomass production in *A. thaliana* and observed a plant growth-promoting effect with concentrations ranging from 50 µM to 150 µM. However, when the treatment surpassed that concentration, there was a significant decrease in primary root length. They hypothesized that 6PP mediates lateral root formation by altering auxin signaling components. This result correlates with the plant fitness data measured seven days after sowing. Seeds treated with 228 mM 6PP had the smallest percentage (58%) of seedlings in category 3 (normal seedlings) and the highest percentage (20%) of seedlings in category 1 (abnormal seedlings).

When 6PP is applied directly to a substrate (filter paper, growth media, potting mix, soil) the amount of 6PP available to the seedling is possibly very high, as it stays in the surroundings of the plant for longer and is in direct contact with the root system, suggesting that the exposure time and proximity are also important when interacting with the plant. Although the optimal exposure duration for 6PP is not yet fully known, our results indicate that seed coating with 6PP can prime seedlings for enhanced growth and drought tolerance. While this concentration slightly reduced seedling emergence, the seedlings that did emerge showed improved growth following drought stress, demonstrating the priming effect of 6PP. The lack of differences in seedling emergence and plant fitness relative to the untreated controls may suggest that the micromolar concentrations of 6PP applied to the seeds were not high enough to induce perceptible morphological changes at germination. Therefore, millimolar concentrations were used in subsequent experiments. The first signal of this interaction between 6PP and the plant was a gradual yet consistent decrease in both seedling emergence and fitness as 6PP concentration increased. The highest 6PP concentration (228 mM) significantly reduced emergence and the number of normal seedlings, providing evidence of an interaction between the 6PP and the seedling.

### 4.2 Measurement of plant growth-related parameters after drought stress

The growth and development of plants are greatly affected by drought, one of the most significant abiotic stresses (Ceccarelli et al., 2004; Ding et al., 2011; Farooq et al., 2012; Kilimani et al., 2018). Plants have developed a wide variety of defense mechanisms to withstand drought, including morphological and structural changes in response to drought stress. Growth inhibition is the principal manifestation of the physiological effects of drought stress. Under ideal conditions, shoots and roots grow in a balanced proportion, and the resource use efficiency is relatively high (Li et al., 2009; Mukherjee et al., 2018). The role of VOCs (whether they are from other plants or microorganisms) in promoting plant growth and activating plant defense mechanisms is now acknowledged. Although the mechanisms are not fully understood, the up-regulation of pathogenesis-related proteins, β -1,3-beta-glucanase, lipoxygenases, peroxidases, and the regulation of the auxin-related pathway have been reported (Agnolucci et al., 2020; Carillo et al., 2020; Chautá and Kessler, 2022; Naznin et al., 2013; Nieto-Jacobo et al., 2017; Phoka et al., 2020; Tahir et al., 2017). Here we provide evidence of the biological role of 6PP as a stress hormone, able to confer advantageous traits to bean plants and enhance drought stress tolerance. In an initial visual examination of the plants after they were subjected to the drought stress and then allowed to recover for ten days, the plants derived from seed treated with 6PP coped better with drought stress (**Figure 3A**), although the plant dry weight after drought stress did not differ from that of the polymer control. However, the fact that the plants maintained turgor suggests that 6PP can exert a positive effect on plants facing drought stress by increasing plant resilience and by boosting plant recovery, possibly due to the production of PPO, PO, β -1,3-beta-glucanase, and chitinase; the production of these molecules has been attributed to the activation of plant defense mechanisms (Conrath et al., 2006; El-Hasan and Buchenauer, 2009; Mauch-Mani et al., 2017).

Plants previously exposed to plant VOCs from stressed plants have been shown to respond more rapidly to biotic and abiotic stressors (Delory et al., 2016; Gfeller et al., 2019; Kessler et al., 2006). The same effect has been observed to a lesser extent when plants are exposed to VOCs produced by beneficial microorganisms (Martínez-Medina et al., 2017; Phoka et al., 2020). To date, it is not clear what exposure time is required for volatiles to exert an effect on plants. While some experiments have only allowed the VOCs to interact with the plant for a couple of hours (Hanson and Kende, 1975; Keegan et al., 1989; Kessler et al., 2006), other experiments extend the time of the VOCs interaction to days (Garnica-Vergara et al., 2016; Phoka et al., 2020). In both cases beneficial effects have been reported e.g., metabolic changes when facing herbivore attack, plant priming against biotic and abiotic stressors, induction of lateral root formation, and stimulation of plant growth (Chautá and Kessler, 2022; Delory et al., 2016; Garnica-Vergara et al., 2016; Kessler et al., 2006; Phoka et al., 2020).

#### 4.2.1 Leaf area after drought stress

Drought stress reduced the total leaf area of plants grown from non-6PP-treated seeds, whereas the plants grown from seeds treated with 6PP had a greater leaf area (51%-68%). These results align with (Sapeta et al., 2013), who observed that, in low water availability, while the growth rate decreased, plants continued to grow during the 28 days of drought stress. This increase is possibly due to the ability of 6PP to induce the production of the defense-related molecules noted above, plus superoxide dismutase, glucanases, gamma-aminobutyric acid (GABA) and acetylcholine, conferring the plant an enhanced capability to cope against drought stress (Braga-Reis et al., 2021; Abbas El-Hasan and Buchenauer, 2009; Mazzei et al., 2016; Sita and Kumar, 2020; Su et al., 2020). The mechanisms by which 6PP stimulates the production of these molecules are still unclear. One possible explanation is that 6PP induces the activation of ISR in plants, which primes the whole plant, triggering a faster and enhanced activation of defense mechanisms under stress conditions. Therefore, the plant can reduce the negative effects of drought stress (Romera et al., 2019). This could explain why the leaves were able to better withstand dehydration than the polymer control. The reduction of total leaf area has been reported as a mechanism that plants utilize for water conservation. This strategy allows plants to minimize the transpiration area (S. Anjum et al., 2011; Boyer, 1970) (Anjum et al., 2017). described the impact of mild and severe drought stress on leaf area. Mild drought is characterized by a decrease in leaf number, leaf expansion rate, and leaf size; while severe drought reduces leaf elongation and stops leaf development. In non-6PP-treated plants, our results agree with those of (Anjum et al., 2017): the leaf area was significantly reduced. However, the plants treated with 6PP continued to grow. This behavior suggests that the application of 6PP via seed coating either induces priming in the plant or gives the plants a growth advantage that cannot be overtaken by the plants grown from non-treated seeds.

#### 4.2.2 Root length after drought stress

There was a significant difference in plant root length after drought stress, especially when the seeds were treated with 114 mM and 228 mM of 6PP. It has been reported that the 6PP molecule displays an auxin-like effect (Garnica-Vergara et al., 2016) and triggers root architectural responses when it interacts with the root system of the plant in a concentration-dependent manner. The increased root length of treated plants, along with the results discussed previously, suggests a correlation between root and leaf area, in which a higher root biomass allows the plant to develop a better shoot system compared to the untreated control. Visual assessment revealed clear morphological differences between treatments under drought stress, as plants grown from 6PP-treated seeds developed a more robust canopy and greater root biomass than those in the polymer control. These differences were still evident 35 days after recovery.

#### 4.2.3 Relative chlorophyll content (SPAD-502)

Drought stress reduces chlorophyll content, thereby reducing the plant’s photosynthetic capacity (S. Anjum et al., 2011, 2017; Sapeta et al., 2013). The plants treated with 114 mM of 6PP showed significantly higher SPAD chlorophyll readings than the untreated control during drought stress. Higher chlorophyll content is closely associated with increased photosynthetic rates and faster leaf expansion, consistent with our observed increases in leaf area, SPAD values, and LI-COR measurements. During drought periods, plants need to maintain efficient photosynthesis to continue to produce energy and survive. In response to these conditions, plants try to increase chlorophyll production to enhance their ability to capture light and drive photosynthesis (Xu et al., 2015). It is important to note that during drought stress, the SPAD readings of the plants treated with 114 mM of 6PP were also significantly higher than those of the polymer and the 75 mM of 6PP treatment, suggesting an enhanced drought stress protection when this concentration is applied via seed coating. The increase in chlorophyll content helps the plant maintain energy production and continue to grow despite water stress. Additionally, chlorophyll can act as an antioxidant, helping protect the plant from the damaging effects of oxidative stress caused by drought and other environmental factors (Xu et al., 2015). At this point, it is reasonable to assume that 6PP may protect plants against abiotic stress, triggering defense mechanisms that lessen the effects of drought in plants derived from 6PP-treated seeds.

#### 4.2.4 Gas exchange response pre-, during, and post-drought stress (LI-COR 6800)

Before drought stress was applied, there were no treatment differences in photosynthetic rates and stomatal conductance, indicating similar physiological status at this stage. This suggests that 6PP does not interfere with normal plant development under non-stress conditions but its effects become evident once the plants are under stress, as indicated by the morphological and physiological responses recorded. Although no differences were detected in photosynthetic rate or stomatal conductance prior to drought imposition, this does not exclude the possibility that 6PP induced underlying enzymatic, hormonal, or metabolomic changes that were not phenotypically expressed at this stage, as previously reported (Jatana et al., 2024; Mondal and Bose, 2022; Yang et al., 2022).

During drought stress, the gas exchange parameters of plants grown from 6PP-coated seeds had a significantly higher stomatal conductance and photosynthetic rates compared to the polymer control. These results suggest that 6PP may facilitate sustained CO_2_ assimilation and gas exchange during water limitation, possibly through improved stomatal regulation or protection of photosynthetic machinery. Such behavior has been associated with primed plants capable of maintaining partial stomatal aperture and active photosynthesis under stress, thereby minimizing irreversible damage to photosynthetic tissues (Sita and Kumar, 2020; Zhang et al., 2015). The 114 mM 6PP seed treatment consistently produced the highest conductance and photosynthetic values, aligning with the enhanced leaf area and relative chlorophyll content (SPAD readings). The mechanisms by which these 6PP induced modifications occur is still unknown. This concentration-dependent effect reinforces the notion that 6PP operates within an optimal physiological range.

Ten days after the drought stress was terminated a partial recovery in gas exchange parameters was evident in all treatments. Stomatal conductance and photosynthetic rate increased across the groups, though the differences between 6PP-treated and polymer control plants were not significantly different. The 114 mM 6PP treatment maintained the highest stomatal conductance post-drought, with a 32% increase when compared to the polymer control. There was also a significantly higher photosynthetic rate when compared to the polymer control. These findings indicate that the 6PP protective activity observed during drought does not persist after stress removal, suggesting that the protective effect may be transient and primarily active only during the stress phase. This is not inherently detrimental, as primed plants will typically exhibit a stronger and more robust response during the stress phase, therefore allocating more energy into modifying their physiological response against such stress and not in growth and development. This primed state ultimately results in a greater gas-exchange response that directly impacts carbon assimilation efficiency, maintenance of photosystem integrity, and overall recovery capacity once favorable conditions are restored. This may allow the plants to resume photosynthesis more rapidly and sustain productivity under fluctuating environmental conditions (Aswathi et al., 2022; Janah et al., 2025; Zaid et al., 2022).

### 4.3 Decoding the transcriptional changes in plants triggered by 6PP seed coating

To date, there is limited knowledge regarding the specific mechanism of action and biological functions associated with 6PP and plants (Carillo et al., 2020; Cutler et al., 1986; El-Hasan and Buchenauer, 2009; El-Hasan et al., 2007; Garnica-Vergara et al., 2016; Mendoza-Mendoza et al., 2024; Taha et al., 2021). This study, therefore aimed to identify changes in molecular pathways, regulatory networks, and biological processes in drought-stressed plants after early exposure to 6PP, in other words, the priming capability of 6PP.

#### 4.3.1 Transcriptional changes induced by 6PP seed coating

The presence of 6PP in well-watered plants increased expression of the CYP707A2-like gene encoding an abscisic acid 8’-hydroxylase. Regulation of ABA 8’-hydroxylase 2 suggests altered ABA catabolism and hormone homeostasis, which may contribute to a primed physiological state under non-stress conditions. In *Arabidopsis*, the CYP707A family regulates ABA levels during seed imbibition and under drought stress (Kushiro et al., 2004). Moreover, other genes upregulated in well water are more related to cytoskeletal remodeling (e.g. PHAVU_002G049700g) and linked to growth and hormone response, especially brassinosteroid-related pathways (e.g. Exordium, PHAVU_008G175800g and PHAVU_008G176000g) (Schröder et al., 2011). Although 6PP is already known to affect auxin transport and root growth (Garnica-Vergara et al., 2016), our results indicate that it may also influence leaf hormone signaling at the transcriptional level. Further studies are needed to determine how this VOC shapes plant physiology under well-watered conditions and whether it acts as a priming signal.

Gene Ontology (GO) categories and KEGG pathways related to photosynthesis and light harvesting were enriched in drought-stressed plants grown from seeds treated with 6PP. During drought stress conditions, CO_2_ assimilation is reduced due to stomatal closure, and subsequent restriction of gas exchange leads to a decline in photosynthetic efficiency (Ali et al., 2025; Ohashi et al., 2006). However, plants grown from 6PP-coated seeds maintained a significantly higher photosynthetic capacity than the polymer control treatment, consistent with the physiological data showing higher gas exchange parameters. This result suggests that this enhanced photosynthetic activity can contribute to stress tolerance by supplying the plant with the essential energy and building blocks required for defense response and recovery from stress (Yang et al., 2021). The significant differential expression of genes encoding chlorophyll-binding proteins (e.g., PHAVU_005G085800G, PHAVU_009G186500G, PHAVU_011G155700G, PHAVU_008G285100G) and components of photosystems I and II supports this observation. These genes are essential for light energy capture and electron transport, enabling sustained ATP and NADPH generation under limited water availability (Chaves et al., 2009; Flexas and Medrano, 2002). These expression patterns suggest that 6PP may help preserve the structural and functional integrity of the photosynthetic apparatus, enabling continued carbon assimilation even under stress. However, the plants grown from 6PP-treated seeds maintained, albeit at a reduced level, a higher photosynthetic capacity than the untreated plants under the same conditions. The identified genes play various roles in photosynthesis and light harvesting in plants. Some of the genes encode chlorophyll-binding proteins, such as PHAVU_005G085800g and the light-harvesting complex I chlorophyll A/B-binding proteins (PHAVU_009G186500g, PHAVU_011G155700g, PHAVU_007G113000g, PHAVU_008G285100g, PHAVU_004G128200g). These proteins capture light energy and transfer it to the reaction centers of photosystems, where it is converted into chemical energy during photosynthesis. Other genes, such as PHAVU_006G216800g, PHAVU_010G128900g, PHAVU_010G012700g, and PHAVU_002G269400g, are associated with electron transfer and redox reactions in photosynthesis. These genes encode proteins involved in electron transport chains, which generate energy-rich molecules such as ATP and NADPH during photosynthesis. Additionally, some of the identified genes encode important enzymes in photosynthesis. For example, PHAVU_001G234300g encodes ribulose bisphosphate carboxylase/oxygenase, which is involved in the activation and regulation of the key enzyme, Ribulose-1,5-bisphosphate (RuBP) carboxylase/oxygenase (RuBisCO), in the Calvin cycle of photosynthesis (Zhan et al., 2022).

The enrichment of phenylpropanoid biosynthesis, which is closely linked to lignin, suggests that under drought stress, 6PP treatment stimulates the production of phenolic compounds, which play an important role in plant defense (Geng et al., 2020; Ninkuu et al., 2025). Phenolic compounds such as flavonoids, lignin, and other secondary metabolites play crucial roles as antioxidants and structural components that protect plants against pathogen attack and UV radiation, mitigate oxidative stress, and reinforce cell walls during dehydration (Lattanzio, 2013; Šamec et al., 2021). The up-regulation of genes involved in phenylpropanoid biosynthesis, such as phenylalanine ammonia-lyase (PHAVU_001G1777001g, PHAVU_001G177800g, PHAVU_007G150500g), cinnamoyl-CoA reductase (PHAVU_005G182100g), caffeoyl-CoA 3-O-methyltransferase (PHAVU_003G047900g), and 4-coumarate-CoA ligase 4 (PHAVU_003G147000g), has an important role in the synthesis of intermediates and enzymes involved in the phenylpropanoid pathway (Šamec et al., 2021). The up-regulation of these genes suggests increased production of phenylpropanoid-related molecules, which can ultimately help the plant reinforce the cell wall and scavenge reactive oxygen species, thereby enhancing the plant’s ability to cope with drought stress (Choi et al., 2023; Geng et al., 2020). The enrichment of phenylpropanoid and flavonoid biosynthesis pathways suggests activation of secondary metabolism; however, transcriptomic data alone do not confirm metabolite accumulation. Future studies incorporating targeted or untargeted metabolite profiling will be necessary to validate these predictions.

The up- and down-regulation dynamics observed in cell wall remodeling enzymes indicate that plants under drought stress shift from wall-loosening to wall-reinforcing processes. The down-regulation of xyloglucan endotransglucosylase/hydrolase (PHAVU_011G085200g), alpha-L-arabinofuranosidase/beta-D-xylosidase (PHAVU_009G254300g), and xyloglucan galactosyltransferase (PHAVU_ 007G063500g) suggests a reduction in cell wall extensibility and growth-related remodeling events (Cosgrove, 2016; Sasidharan et al., 2011). On the other hand, the up-regulation of cellulose synthase genes (PHAVU_005G116200g, PHAVU_005G116500g, PHAVU_006G058400g) supports the hypothesis that 6PP treatment enhances cellulose deposition and wall reinforcement. Increased cellulose biosynthesis is a common drought-adaptive mechanism that strengthens cell walls and prevents mechanical collapse under water deficit.

Further analysis of genes differentially expressed during drought stress revealed that plants grown from 6PP-treated seeds showed enrichment in GO categories associated with protein phosphorylation. This enrichment is closely linked to the activation of various signaling pathways, including those critical for plant defense responses, such as MAP-kinase, calcium-dependent protein kinase, and phytohormone signaling pathways (Gilroy and Breen, 2022; Niraula et al., 2020; Shi et al., 2018). The up-regulation of genes associated with protein kinases, such as receptor-like kinases and serine/threonine kinases (PHAVU_008G030400g, PHAVU_008G030800g, PHAVU_004G039200g, PHAVU_008G211200g, PHAVU_005G162600g, PHAVU_001G043000g), which play a key role in activating the phosphorylation-mediated signaling cascade, was identified. Their enhanced expression suggests an increased cellular capacity to perceive and propagate stress signals, thereby regulating essential plant defense processes, including hormone signaling, pathogen recognition, and general stress responses (Zhang et al., 2021). The transcriptomic evidence was further supported by the MAPK phosphorylation assay, which demonstrated that MAPK phosphorylation increased under drought stress and was further enhanced in plants treated with 6PP compared with the polymer control. Although a low level of MAPK phosphorylation was detected under well-watered conditions, the strongest phosphorylation signal was observed in drought-stressed plants treated with 6PP (**Figure 8**). MAPK cascades are central components of plant stress signaling and are rapidly activated in response to environmental challenges. The enhanced phosphorylation observed in 6PP-treated plants under drought provides protein-level evidence that complements the transcriptomic enrichment of phosphorylation-related pathways, indicating that the transcriptional changes identified are accompanied by activation of downstream signaling cascades. This enhanced MAPK activation likely contributes to the coordinated regulation of defense-related genes, transcription factors, and physiological responses observed in 6PP-treated plants, supporting the hypothesis that 6PP potentiates plant stress signaling and promotes a more effective response to drought stress.

These findings may indicate that 6PP is involved in the induction of a priming state because the expression of these genes suggests an enhanced ability of the plant to perceive and respond efficiently to stress signals. In addition, the up-regulation of defense-related proteins, such as chitinases (PHAVU_011G167300g), glucanases (PHAVU_011G177000g, PHAVU_011G077900g), and peroxidases (PHAVU_007G107400g), further supports the previous findings and the hypothesis that the 6PP treatment enhances the plant’s defense response under drought stress. While chitinases and glucanases are well known for their involvement in plant response against pathogens, they have been also associated with abiotic stress responses, often through roles in cell wall remodeling or as signaling molecules that activate defense pathways (Gregorova et al., 2015). Peroxidases, on the other hand, are commonly associated with several processes, including cell wall strengthening, lignin biosynthesis, ROS scavenging, and abiotic stress response. They play a crucial role in cell wall reinforcement because of their involvement in lignification, a process where they catalyze the polymerization of monolignols to form lignin, which provides mechanical strength and resistance against stresses like drought (Goche et al., 2020; Gu et al., 2020; Yadav and Chattopadhyay, 2023). In addition, peroxidases are central to the plant’s defense against oxidative damage by detoxifying harmful ROS, such as hydrogen peroxide, which accumulate under abiotic stress conditions (Romera et al., 2019). All these genes together play an important role in plant defense against both biotic and abiotic stresses, including drought. Consistent with the activation of defense responses and signaling pathways previously discussed, an up-regulation of transcription factors was observed in plants grown from seeds treated with 6PP. Specifically, members of the WRKY family (PHAVU_001G042100g, PHAVU_008G081800g), NAC transcription factors (PHAVU_011G024700g, PHAVU_006G188900g, PHAVU_011G147800g), AP2/ERF factors (PHAVU_007G082000g, PHAVU_010G130200g), MYB proteins (PHAVU_008G236500g), CONSTANS-like zinc-finger factors (PHAVU_003G149000g, PHAVU_004G157300g), heat shock transcription factors (PHAVU_002G019100g), as well as additional bHLH and homeobox/HD-ZIP proteins (e.g. PHAVU_008G087200g, PHAVU_004G028600g, PHAVU_009G049600g) were induced in response to drought in the 6PP treatment. These transcription factor families are recognized as important regulators of abiotic stress and priming responses, hormonal (ABA, JA, ethylene, SA) and ROS signals with downstream expression of defense-related and metabolic genes, including those involved in osmotic adjustment, antioxidant capacity, and cell-wall and secondary-metabolite biosynthesis (Chowdhary et al., 2023; Nuruzzaman et al., 2013; Wu et al., 2024; Zheng et al., 2025). The up-regulation of these transcription factors suggests that 6PP treatment induces a broad transcriptional reprogramming, leading to coordinated gene expression involved in stress tolerance, hormone signaling, and defense-related metabolite production, consistent with a prime state of the plant.

This analysis of GO enrichment categories and genes provides the first detailed insight into the transcriptional changes induced by 6PP seed-coating treatment in plants grown under drought stress. By improving photosynthesis, stimulating phenylpropanoid biosynthesis, activating signaling pathways, and positively enhancing gene expression under unfavorable conditions, 6PP has been demonstrated to have an active role in protecting plants. Furthermore, the behavior of the treated plants under drought stress conditions suggests a priming state induced by the 6PP.

## 5 Conclusions

This study aimed to assess the biological significance of 6PP and its ability to enhance plant resilience to drought stress by inducing plant priming through seed coating as the delivery method. The results confirm a concentration-dependent effect of 6PP: high concentrations can impair seedling emergence and fitness, while low concentrations may not produce the desired responses. 6PP, therefore, has potential as a tool for promoting plant growth and health in agriculture and horticulture through its ability to positively impact seedling emergence, plant fitness, and chlorophyll relative content in drought-stressed plants. 6PP induced the expression of defense-related genes, which may stimulate the plant’s defense mechanisms. However, more research is needed to fully understand the molecular mechanisms involved and determine the optimal concentration for different plant species. Overall, the dwarf bean plants grown from 6PP-treated seeds showed different gene expression dynamics compared to those observed in the control treatment. The transcriptomic analysis of drought-stressed plants derived from 6PP-treated seeds revealed enrichment of GO categories related to photosynthesis, phenylpropanoid biosynthesis, signaling, and defense responses. These enriched categories appear to be the plants’ mechanism for defending against unknown challenging environmental conditions, indicating the use of general defense mechanisms to cope with drought stress. Despite a decreased photosynthetic rate under drought stress, plants grown from 6PP-treated seeds had a higher photosynthetic efficiency than untreated plants.

Furthermore, genes involved in phenylpropanoid biosynthesis, which aid in coping with various abiotic stresses such as salinity, extreme temperatures, UV radiation, heavy metals, and drought, were identified. The up-regulation of protein phosphorylation-related genes and several transcription factor genes suggested the activation of signaling pathways involved in plant defense response through transcriptional reprogramming. Further examination and characterization of these genes will help determine whether 6PP can trigger a priming state in the plant and provide insights into the potential pathways involved.

## Data Availability

The data presented in the study are deposited in the GEO repository, accession number GSE339170, https://www.ncbi.nlm.nih.gov/geo/.
