# Supplementary material for: The fungal volatile organic compound 6-pentyl-alpha-pyrone primes dwarf bean for enhanced drought tolerance
Source: Front Plant Sci. 2026 Aug 12;17:1833542. doi: 10.3389/fpls.2026.1833542 (PMC13507937; doi:10.3389/fpls.2026.1833542)
Supplement: Supplementary file 1 [file SupplementaryFile1.zip › Supplementary Material/Supplementary Figures.docx]

## 1.1 Supplementary Figures

**Supplementary Figure S1. Measurement of relative chlorophyll content using a SPAD meter.**
Relative chlorophyll content was measured on the second fully developed trifoliate leaf from the top of the plant using a SPAD chlorophyll meter (Konica Minolta, Japan). SPAD readings were taken from three positions on the leaflet: near the base, at the midpoint between the base and the tip, and near the tip (indicated by red dots). The average of these readings was used to represent the relative chlorophyll content.

**Supplementary Figure S2. Leaf area measurement using Easy Leaf Area software.** **(A)** Original photograph of a trifoliate leaf placed on a white background with a 4 cm² red reference square used for scale calibration. **(B)** Processed image after colour threshold adjustment and background removal in Easy Leaf Area, where the detected leaf area is highlighted in green and the software calculates the total leaf area based on the reference square.
