# Supplementary material for: The fungal volatile organic compound 6-pentyl-alpha-pyrone primes dwarf bean for enhanced drought tolerance
Source: Front Plant Sci. 2026 Aug 12;17:1833542. doi: 10.3389/fpls.2026.1833542 (PMC13507937; doi:10.3389/fpls.2026.1833542)
Supplement: Supplementary file 1 [file SupplementaryFile1.zip › Supplementary Material/Supplementary Table 1.docx]

| **Supplementary Table 1.** Differentially expressed genes in well-watered plants exposed to 6-PP compared with the polymer control | | | | |
| --- | --- | --- | --- | --- |
| Gene symbol | Locus tag | Gene description | Log₂ fold change | Bonferroni |
| PHAVU_008G205100g | LOC137824164 | Urease accessory protein F | 3.16 | 2.17E-04 |
| PHAVU_008G175800g | LOC137824469 | Exordium-like protein | 2.07 | 2.70E-07 |
| PHAVU_008G176000g | LOC137824312 | Exordium-like protein | 1.51 | 3.15E-03 |
| PHAVU_002G049700g | LOC137810415 | Formin-like protein 18 | 1.40 | 2.96E-05 |
| PHAVU_002G122200g | LOC137811095 | Abscisic acid 8'-hydroxylase | 1.37 | 3.31E-06 |
| rps3 | GH805_mgp03 | Ribosomal protein S3 | 1.32 | 9.02E-03 |
| rps2 | PhvuCp25 | Ribosomal protein S2 | 1.22 | 4.53E-03 |
| PHAVU_005G027300g | LOC137836130 | Proline-rich cell wall protein 3-like | 1.21 | 7.02E-07 |
| PHAVU_009G090248g | LOC137822243 | uS12 (small ribosomal subunit protein) | 1.19 | 3.85E-02 |
| LOC137817479 | LOC137817479 | Senescence-associated protein | 1.09 | 7.74E-11 |
| rps3 | PhvuCp01 | Ribosomal protein S3 | 1.06 | 2.00E-03 |
| PHAVU_001G088700g | LOC137813646 | CWF21 domain-containing protein | -0.63 | 3.15E-02 |
| PHAVU_010G091800g | LOC137818315 | LRR disease resistance protein | -0.85 | 3.72E-03 |
| PHAVU_001G112100g | LOC137813937 | Uncharacterized protein | -0.88 | 8.84E-06 |
| PHAVU_008G252000g | LOC137826521 | Albumin-2 | -0.88 | 1.68E-03 |
| PHAVU_007G275700g | LOC137827554 | Albumin-2 | -0.92 | 8.21E-03 |
| PHAVU_007G091400g | LOC137828482 | Glycine-rich cell wall protein 1 | -0.98 | 4.81E-09 |
| PHAVU_002G121700g | LOC137811089 | Rhodopsin | -1.05 | 7.33E-09 |
| LOC137817722 | LOC137817722 | Uncharacterized LOC137817722 | -1.13 | 2.70E-02 |
| PHAVU_007G277000g | LOC137827563 | Albumin-2 | -1.43 | 2.00E-02 |
| PHAVU_008G266800g | LOC137825705 | Reactive intermediate deaminase A | -1.10 | 1.13E-10 |
| atp1 | GH805_mgp08 | ATPase subunit 1 | 1.00 | 4.50E-02 |
| psbA | PhvuCp04 | Photosystem II protein D1 | 1.34 | 1.66E-11 |
| rbcL | PhvuCp06 | Ribulose-1,5-bisphosphate carboxylase | 1.00 | 2.10E-04 |
| atpB | PhvuCp07 | ATP synthase CF1 beta subunit | 1.19 | 1.25E-07 |
| atpE | PhvuCp08 | ATP synthase CF1 epsilon subunit | 1.31 | 7.13E-07 |
| psaA | PhvuCp14 | Photosystem I P700 apoprotein A1 | 1.26 | 2.15E-10 |
| psaB | PhvuCp15 | Photosystem I P700 chlorophyll a apoprotein A2 | 1.37 | 1.98E-14 |
| atpI | PhvuCp26 | ATP synthase CF0 A subunit | 1.33 | 1.54E-11 |
| atpF | PhvuCp28 | ATP synthase CF0 B subunit | 0.87 | 1.25E-02 |
| atpA | PhvuCp29 | ATP synthase CF1 alpha subunit | 0.88 | 1.10E-02 |
| ycf4 | PhvuCp35 | Photosystem I assembly protein Ycf4 | 0.93 | 3.65E-06 |
| cemA | PhvuCp36 | Envelope membrane protein | 0.64 | 1.89E-02 |
| psbB | PhvuCp50 | Photosystem II 47 kDa protein | 1.20 | 3.32E-04 |
| petB | PhvuCp54 | Cytochrome b6 | 1.65 | 5.37E-04 |
| psaC | PhvuCp66 | Photosystem I subunit VII | 2.11 | 3.67E-04 |
|  | LOC137816771 | 18S ribosomal RNA | 2.20 | 1.15E-04 |
|  | LOC137808852 | 28S ribosomal RNA | 1.62 | 2.26E-04 |
|  | LOC137817470 | 28S ribosomal RNA | 1.81 | 8.06E-24 |
|  | LOC137817483 | 18S ribosomal RNA | 1.42 | 1.20E-05 |
|  | LOC137817481 | 18S ribosomal RNA | 3.64 | 1.48E-03 |
|  | LOC137817484 | 28S ribosomal RNA | 1.24 | 6.24E-14 |
|  | LOC137817490 | 18S ribosomal RNA | 0.90 | 2.94E-03 |
|  | LOC137817729 | 28S ribosomal RNA | 2.64 | 1.30E-06 |
|  | rrnS | Small subunit ribosomal RNA | 1.04 | 3.77E-05 |
|  | rrn16 | 16S ribosomal RNA | 1.52 | 4.31E-15 |
|  | rrn23 | 23S ribosomal RNA | 1.39 | 5.81E-09 |
|  | rrn23 | 23S ribosomal RNA | 1.39 | 5.49E-09 |
|  | rrn16 | 16S ribosomal RNA | 1.51 | 5.80E-15 |
